# Tropical tree species with high wood specific gravity have higher concentrations of wood phosphorus and are more efficient at resorbing it

**DOI:** 10.1093/aobpla/plaf001

**Published:** 2025-01-03

**Authors:** Andrés González-Melo, Juan Manuel Posada, Jacques Beauchêne, Romain Lehnebach, Bruno Clair

**Affiliations:** Biology Department, Faculty of Natural Sciences, Universidad del Rosario, Avenida carrera 24 # 63C-69, Bogotá 110111, Colombia; Biology Department, Faculty of Natural Sciences, Universidad del Rosario, Avenida carrera 24 # 63C-69, Bogotá 110111, Colombia; CIRAD, UMR Ecologie des Forêts de Guyane (EcoFoG), AgroParisTech, CNRS, INRAE, Université des Antilles, Université de Guyane, 97310 Kourou, France; CIRAD, UMR Ecologie des Forêts de Guyane (EcoFoG), AgroParisTech, CNRS, INRAE, Université des Antilles, Université de Guyane, 97310 Kourou, France; CNRS, UMR Ecologie des Forêt de Guyane (EcoFoG), AgroParisTech, CIRAD, INRAE, Université des Antilles, Université de Guyane, 97310 Kourou, France; Laboratoire de Mécanique de Génie Civil (LMGC), CNRS, Université de Montpellier, 34000 Montpellier, France

**Keywords:** wood nutrients, wood specific gravity, wood anatomy, tropical trees, nutrient resorption

## Abstract

Phosphorus (P) and potassium (K) play important roles in plant metabolism and hydraulic balance, respectively, while calcium (Ca) and magnesium (Mg) are important components of cell walls. Although significant amounts of these nutrients are found in wood, relatively little is known on how the wood concentrations of these nutrients are related to other wood traits, or on the factors driving the resorption of these nutrients within stems. We measured wood nutrient (i.e. P, K, Ca, and Mg) concentrations, wood specific gravity (WSG), as well as wood fibre and parenchyma fractions, in both inner (i.e. close to the pith) and outer (i.e. close to the bark) wood, for 22 tree species from a rainforest of eastern Amazonia. We first examined the associations of wood nutrient concentrations with WSG, fibre fractions, and parenchyma fractions. Then, we assessed whether resorption rates (i.e. difference between heartwood and sapwood nutrient contents) differed among nutrients, and whether nutrient resorption rates were related to species ecological strategies. WSG was unrelated to wood Ca, positively related to wood P in outer wood, and negatively related to inner wood Mg, as well as to both inner and outer wood K. Overall, nutrients were unrelated or negatively related to fibre and parenchyma fractions, except for wood Ca and wood P, which were positively related to fibre and axial parenchyma fractions in outer wood, respectively. We found that resorption rates did not differ among nutrients, and that P resorption rates were higher in high WSG, while K, Ca, and Mg resorption rates were unrelated to WSG. This study illustrates that the relationships of wood nutrient concentration with WSG and cell type fractions can be nutrient-specific. Our results indicate that, excluding a positive association between wood Ca and fibre fractions, and between wood P and axial parenchyma fractions, wood nutrients were mostly unrelated to anatomical traits. Our findings also suggest that high-WSG (i.e. shade-tolerant) species store higher amounts of wood P, and are more efficient at resorbing wood P, than low-WSG (i.e. fast-growing) species. These insights are important to increase our understanding on wood nutrient allocation, nutrient resorption, and tree ecological strategies in lowland tropical forests.

## Teaser

This study shows that the relationships of wood nutrients with wood specific gravity and wood anatomical traits can be nutrient-specific in a lowland tropical rainforest. Likewise, it illustrates that tropical tree species with high wood specific gravity tend to have higher concentrations of wood phosphorus, and are better at resorbing it from inner to outer wood.

## Introduction

Phosphorus (P) and base cations such as potassium (K), calcium (Ca), and magnesium (Mg) are essential nutrients for plant functioning (e.g. Vitousek 1984; Chapin *et al.* 2011). Phosphorus, e.g., plays a central role in plant metabolism and can be limiting in some plant communities such as lowland tropical forests (Vitousek 1984; Dalling *et al.* 2016; Santiago and Goldstein 2016; Jiang *et al.* 2019). Potassium, on the other hand, is involved in hydraulic balance (e.g. Sardans and Peñuelas 2015), while Ca and Mg act as structural components of cell walls (e.g. Lautner *et al.* 2007). In forest ecosystems, a significant amount of these nutrients is in wood (Heineman *et al.* 2016; Bauters *et al.* 2022; Inagawa *et al.* 2023), which represents in some cases up to 50% of these nutrients in forest stocks (e.g. Tanner 1985). Wood is, therefore, a key component of nutrient strategies among forest plant species (Heineman *et al.* 2016). Yet, although significant advances have been made in recent years in quantifying wood P, K, Ca, and Mg concentrations (e.g. Heineman *et al.* 2016; Lira-Martins *et al.* 2019; Bauters *et al.* 2022; Inagawa *et al.* 2023), and in linking them to tree ecological strategies (e.g. Heineman *et al.* 2016; Kotowska *et al.* 2020; Lira-Martins *et al.* 2022), different aspects of wood P, K, Ca, and Mg strategies remain poorly understood. In particular, it is still unclear how the wood concentrations of these nutrients covary with other wood traits, or the factors driving their resorption within woody stems.

The wood economics spectrum (WES) is an important framework linking wood structure with individual functioning and species ecological strategies (Chave *et al.* 2009). Wood specific gravity (WSG), which reflects the carbon investment per unit volume of stem, is a central trait in the WES as it is overall a good indicator of wood properties and plant performance (Muller-Landau 2004; Poorter *et al.* 2008; Chave *et al.* 2009; Wright *et al.* 2010; Visser *et al.* 2016). Woody plant species fall along a continuum of wood structure going from fast-growing species with low WSG to shade-tolerants that typically have high WSG (Chave *et al.* 2009). A main idea derived from the WES is that wood traits covary with WSG (Chave *et al.* 2009). The patterns of covariation between WSG and wood anatomical traits (e.g. Zanne *et al.* 2010; Fortunel *et al.* 2013; Zieminska *et al.* 2015), or among WSG and wood carbon concentrations (e.g. Chave *et al.* 2009; Martin and Thomas 2015; Martin *et al.* 2018), are relatively well studied. In contrast, few studies have investigated how wood P, K, Ca, and Mg concentrations are related to WSG, and they show contradictory results (Heineman *et al.* 2016; Kotowska *et al.* 2020; Inagawa *et al.* 2023). For instance, Inagawa *et al.* (2023) found that WSG was negatively related to wood P and K, positively to wood Ca, and unrelated to wood Mg. Heineman *et al.* (2016), on the other hand, reported that WSG was inversely related to wood P, and unrelated to K, Ca, and Mg, while Kotowska *et al.* (2020) found no relationship between WSG and wood P. These contrasting patterns suggest that more information is needed to elucidate the variation of wood P, K, Ca, and Mg concentrations along the WES.

As specific gravity is an emergent property of wood that is affected by the morphology and relative abundance of different cell types (Carlquist 2001), the variation of wood P, K, Ca, and Mg concentrations along the WES may be better understood by considering how these nutrients are allocated among wood cells. In wood, a substantial fraction of some nutrients, such as Ca and Mg, tends to be allocated to cell walls (Lautner *et al.* 2007). As high-WSG species typically have larger fractions (i.e. % of cross-sectional area) of fibres (e.g. Zieminska *et al.* 2015), they are expected to have higher concentrations of Ca and Mg in comparison to low-WSG species. By contrast, most wood P and K are thought to be stored in parenchyma cells (Merril and Cowling 1966; Meerts 2002). Since low-WSG species generally have higher total parenchyma fractions (e.g. Martínez-Cabrera *et al.* 2009; Zieminska *et al.* 2015), they may store larger amounts of P and K than high-WSG species. In some species, P and K may also be stored in living fibres which are frequent in species with scanty or absent parenchyma and are suggested to play an important role in storage (Carlquist 2015; Plavcová *et al.* 2016, 2023). Although linking wood nutrient concentrations with wood anatomy may provide a more detailed understanding on how these nutrients vary among species and along the WES, studies on this topic have been scarce (Merril and Cowling 1966; Kotowska *et al.* 2020).

Wood nutrient concentrations can vary not only across species with different wood structures, but also radially (i.e. from heartwood to sapwood) within stems (e.g. Meerts 2002; Millard and Grelet 2010; Heineman *et al.* 2016; Inagawa *et al.* 2023). In general, nutrient concentrations tend to be higher in sapwood than in heartwood (reviewed by Meerts 2002), which has been interpreted in several studies as evidence that woody plants resorb nutrients to sapwood once heartwood formation begins (Millard and Grelet 2010; Sette *et al.* 2013; Heineman *et al.* 2016). Nutrient resorption is considered as an important mechanism increasing nutrient use efficiency, particularly in plant communities in nutrient-limited soils (Hill 1980; Urbina *et al.* 2021). Three aspects, which are not mutually exclusive, may influence nutrient resorption rates. First, the functional roles of nutrients affect their resorption rates (Urbina *et al.* 2021). For example, the role of Ca and Mg in cell walls (Lautner *et al.* 2007) means that they are likely to be less mobile than P or K, which are mainly stored in parenchyma cells and can be easily mobilized to be used in metabolism (e.g. Meerts 2002). Consequently, Ca and Mg are generally resorbed at lower rates than P and K (Sette *et al.* 2013; Urbina *et al.* 2021). Second, it has been suggested that the resorption of limiting nutrients should be greater than those of non-limiting ones (McGroddy *et al.* 2004; Posada and Schuur 2011; Sardans and Peñueñas 2015). For instance, since tropical lowland forests tend to be P-limited (Vitousek 1984; Dalling *et al.* 2016), one may expect that in these forests P would be resorbed at higher rates in comparison to other nutrients (Sardans and Peñuelas 2014; Heineman *et al.* 2016). Third, nutrient resorption is suggested to be related to species ecological strategies (Urbina *et al.* 2021). In particular, it is thought to be especially common among high-WSG, shade-tolerant species because they live longer and will have to counteract nutrient limitations, when present, for longer periods in comparison to most low-WSG, fast-growing species (Sardans and Peñuela 2014).

In this study, we quantified wood nutrient (i.e. P, K, Ca, and Mg) concentrations, related them to other wood traits (i.e. WSG, as well as fibre and parenchyma fractions), and assessed their resorption rates for 22 tree species from a lowland rainforest in eastern Amazonia. In particular, we wanted to answer the following questions: (i) How are wood nutrient concentrations related to WSG, fibre fractions, and parenchyma fractions? We hypothesized that the concentrations of wood P and K would be greater in species with larger parenchyma fractions, as these nutrients are expected to be deposited mainly in parenchyma cells. Likewise, we expected both wood P and K to be negatively related with WSG, as high WSG tends to have lower parenchyma fractions. On the other hand, we anticipated that wood Ca and Mg concentrations would scale with fibre fractions, as Ca and Mg are thought to be allocated to cell walls, and fibres are the most abundant cell type in wood angiosperms and have thicker walls than the other cell types. Also, we expected that both wood Ca and Mg would be positively related with WSG, since high-WSG species typically have higher fibre fractions and thicker fibres. (ii) How do resorption rates vary among nutrients, and are nutrient resorption rates related to species ecological strategies? Since K and P tend to be more mobile than Ca and Mg, we expected higher resorption rates (i.e. higher nutrient concentrations in sapwood than in heartwood) of K and P in comparison to Ca and Mg. Higher resorption rates of P are also expected because P is thought to be a limiting nutrient at our study site, and resorption rates are suggested to be higher for limiting nutrients. Finally, we anticipated that nutrient resorption rates will be higher for high-WSG, shade-tolerant species because they live longer and would have to counteract nutrient limitation for longer periods.

## Materials and methods

### Study site

The study was conducted in the Paracou research station in northern French Guiana (5°18ʹN, 52°55ʹW). Mean annual temperature in Paracou is 28.4°C. Annual rainfall averages *c*. 3.000 mm with a marked dry season from August to November, and a distinct rainy season between March and June (Wagner *et al.* 2011). Vegetation at Paracou is lowland tropical rainforest, representative of northeastern Amazonia (ter Steege *et al.* 2006), and the Guiana Shield (ter Steege *et al.* 2000) in terms of diversity and floristic composition. The landscape is dominated by moderate hills separated by narrow streams (Ferry *et al.* 2010). Evidence suggests that soils at Paracou are strongly P-limited. First, these soils, as most soils of the Guiana Shield (Hammond 2005), are ancient and have been subjected to long-term weathering of primary minerals during soil development (Urbina *et al.* 2021). Second, stocks of P in soils, leaves, and litter in Paracou are low (Quesada *et al.* 2012; Urbina *et al.* 2021). Third, P resorption rates from senescing leaves are high at Paracou (Uribina *et al.* 2021), which can be interpreted as evidence of P limitation (e.g. Sardans and Peñuelas 2015).

### Species and sampling

Based on 23-year demographic data from forest permanent plots (Gourlet-Fleury *et al.* 2004; https://paracou.cirad.fr/website/station), we selected 22 tree species that represent a wide range of shade-tolerance and growth rates, and belong to some of the most dominant families at Paracou, namely Fabaceae, Lecythidaceae, and Chrysobalanaceae (Table 1; Baraloto *et al.* 2012). Overall, we sampled 75 mature trees, with two to five individuals per species (Table 1). Most wood samples were collected in Paracou, except for *Cordia alliodora*, *Schefflera morototoni*, *Cecropia obtusa*, and *Miconia tschudyoides*, which were collected in nearby secondary forests on comparable soils. Samples of 10 species (i.e. all legume species, *C. alliodora* and *Bagassa guianensis*) were collected and used in previous studies (Bossu *et al.* 2016; Lehnebach *et al.* 2019).

**Table 1.** Study species, family, number of trees sampled (*n*), mean diameter at breast height of trees sampled (DBH, standard deviation in brackets), ecological guilds, and heartwood presence for 22 Amazonian tree species from a lowland forest in eastern Amazonia.

Species	Family	*n*	DBH (cm)	Ecological guild	Heartwood presence
*Bagassa guianensis*	Moraceae	5	25.1 (2.53)	Semi-tolerant & Canopy	Yes
*Bocoa prouacensis*	Fabaceae	4	12.3 (1.87)	Shade-tolerant & Understory	Yes
*Cecropia obtusa*	Urticaceae	3	16.1 (0.89)	Fast-growing & Understory	No
*Cordia alliodora*	Cordiaceae	4	26.4 (1.86)	Fast-growing & Canopy	Yes
*Dicorynia guianensis*	Fabaceae	5	18.2 (2.41)	Semi-tolerant & Canopy	Yes
*Eperua falcata*	Fabaceae	5	19.5 (2.54)	Semi-tolerant & Canopy	Yes
*Eperua grandiflora*	Fabaceae	3	12.4 (3.55)	Semi-tolerant & Canopy	Yes
*Eschweilera coriacea*	Lecythidaceae	3	16.3 (2.64)	Shade-tolerant & Canopy	No
*Eschweilera sagotiana*	Lecythidaceae	2	17.8 (2.92)	Shade-tolerant & Canopy	No
*Hirtella glandulosa*	Chrysobalanaceae	2	15.3 (3.12)	Shade-tolerant & Understory	No
*Lecythis persistens*	Lecythidaceae	5	16.3 (3.52)	Shade-tolerant & Understory	Yes
*Licania alba*	Chrysobalanaceae	4	14.7 (2.37)	Shade-tolerant & Canopy	Yes
*Miconia tschudyoides*	Melastomataceae	2	13.2 (1.37)	Fast-growing & Understory	No
*Oxandra asbeckii*	Annonaceae	4	14.6 (2.36)	Shade-tolerant & Understory	Yes
*Parkia nitida*	Fabaceae	4	18.5 (3.78)	Fast-growing & Canopy	No
*Parkia velutina*	Fabaceae	3	12.4 (3.14)	Semi-tolerant & Canopy	No
*Peltogyne venosa*	Fabaceae	3	13.1 (2.62)	Shade-tolerant & Understory	Yes
*Recordoxylon speciosum*	Fabaceae	3	12.3 (1.60)	Semi-tolerant & Canopy	Yes
*Schefflera morototoni*	Araliaceae	3	21.2 (3.22)	Fast-growing & Canopy	No
*Sextonia rubra*	Lauraceae	3	22.4 (2.74)	Semi-tolerant & Canopy	Yes
*Swartzia panacoco*	Fabaceae	3	12.5 (2.16)	Shade-tolerant & Canopy	Yes
*Virola michelii*	Myristicaceae	3	20.5 (3.86)	Fast-growing & Canopy	Yes

Ecological guilds were assigned based on Favrichon (1994).

### Wood trait measurements

Wood samples were collected from stem discs taken, at breast height, from trees that had been cut down. Two wood segments (*c*. 2 × 2 × 2 cm) were cut from each stem disc: one at 2 cm from the pith (hereafter called *inner wood*), and the other at 2 cm from the bark (hereafter referred to as *outer wood*). In 14 of the study species (see Table 1), sapwood and heartwood were distinguished based on colour differences (i.e. heartwood is typically dark-coloured). For these species, inner wood corresponds to heartwood, and outer wood to sapwood. All traits (i.e. WSG, wood nutrients, and wood anatomical traits) were measured on both inner and outer wood. For each wood segment, we measured fresh volume and dry mass. Fresh volume was measured by the water displacement method, and dry mass was obtained after drying the segments at 103°C to a constant weight for 72 h. WSG per segment was defined as dry weight over fresh volume (Kollman and Coté 1968).

**Figure 1.**
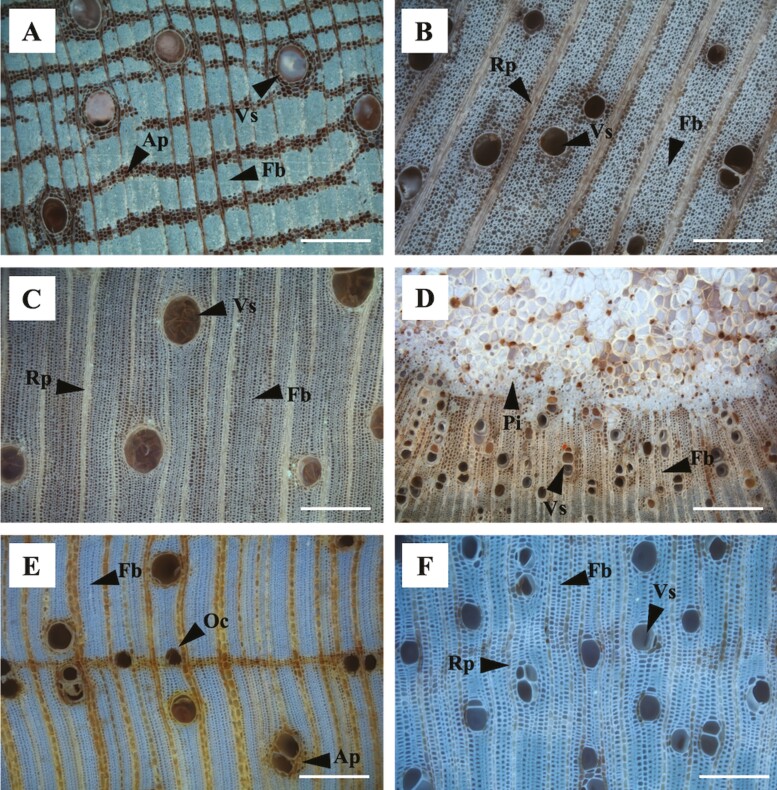
Cross-section anatomical images of six of the study species. (A) *Dicorynia guianensis*, (B) *Cordial alliodora*, (C) *Bagassa guianensis*, (D) *Recordoxylon speciosum*, (E) *Eperua falcata*, and (F) *Miconia tschudyoides*. Scale bars correspond to 200 μm. Ap: axial parenchyma; Rp: radial parenchyma, Fb: Fibres; Vs: vessels; Oc: oil canals; and Pi: pith.

For anatomical analyses, cross-sectional surfaces were sanded using a polishing machine with 1200-grit diamond discs, and then samples were cut with a GLS-1 sledge microtome (Gärtner *et al.* 2015) to get a plane surface. Photographs were taken at 5–10× objective lenses using a reflected light (episcopic) microscope (see Fig. 1; BFMX, Olympus, Tokyo, Japan), equipped with a digital camera (Canon EOS T6i; Canon Inc., Tokyo, Japan). For each wood segment, between 10 and 20 partially focused images were taken and combined using Helicon Focus (see Fig. 1; Helicon Focus Ltd, Kharkov, Ukraine). It was not possible to obtain high-quality anatomical images of *Bocoa prouacensis* because of its very high WSG; thus, this species was excluded from the analysis on parenchyma fractions. Fractions of fibre, axial, and radial parenchyma cells per cross-sectional area were obtained from the anatomical images. To calculate these fractions, fibres and parenchyma cells were first manually coloured using Photoshop (Adobe Systems Incorporated, USA), and then fractions were calculated automatically using a batch function in the software ImageJ (https://imagej.nih.gov/ij/). Three of our study species have living fibres (i.e. *Shefflera morototoni*, *Sextonia rubra*, and *C. obtusa*) that we did not quantify. Thus, for these species fibres fractions reflect both living and dead fibres. After taking the anatomical images, wood segments were ground and ashed at 550°C for 1 h, and then the ash was dissolved in 1 M HNO_3_. Wood P, K, Ca, and Mg concentrations were quantified using inductively coupled plasma-optimal emission spectrometry on an Optima 7300 DV (Perkin-Elmer Ltd, Shelton, CT, USA), with apple leaves (NIST 1515) as a certified reference sample.

### Statistical analyses

We first compared inner and outer wood nutrient concentrations, pooling species together, with *t*-tests. Then, to assess the relationships of wood nutrients with WSG, fibre fractions, and parenchyma fractions, we used standardized major axis (SMA) regressions in the ‘smart’ package for R (Warton *et al.* 2006), using species means as data points. We conducted separate SMA for inner and outer wood data, and the differences in the SMA slopes between inner and outer wood data, when both slopes were significant (*P* < .05), were examined using likelihood ratio tests (LRT). Wood nutrient data and WSG were log-transformed to meet normality assumptions. To compare resorption rates among nutrients, as well as to assess whether nutrient resorption rates were related to species ecological strategies, we considered only the species (*n* = 14) for which we distinguished heartwood from sapwood based on colour differences (see Table 1), given that nutrients are typically resorbed from senescing tissues (e.g. Meerts 2002). We calculated nutrient resorption rates for all species pooled, as well as for each species, as: (outer − inner)/outer × 100 (Heineman *et al.* 2016), where outer and inner are wood nutrient concentrations in sapwood and heartwood, respectively. To compare resorption rates among nutrients we used an analysis of variance. In order to analyse whether nutrient resorption rates are related to species ecological strategies, we used WSG as a proxy of tree ecological strategies, since it is a trait closely related to carbon investments per unit of wood volume, tree functioning, and demography (Carlquist 2001; Chave *et al.* 2009; Wright *et al.* 2010). The relationships of nutrient resorption rates with WSG were examined using linear models, with species means data points, in the ‘car’ package for R (Fox and Weisberg 2011). In these models, the residuals were checked for normal distribution and homoscedasticity using diagnosis plots. All statistical analyses were performed in R 3.6.1 (R Development Core Team 2019).

## Results

Wood nutrient concentrations varied considerably among species (Table 2). The least variable nutrient in inner (15.2-fold) and outer wood (4.67-fold variation) was P, whereas the most variable was K (with 54.3 and 33.1-fold variation, respectively). When all species were pooled together, wood P concentrations were higher in outer than in inner wood (*P* < .001; *t*: 4.00), while the concentrations of K (*P* > .05; *t*:1.35), Ca (*P* > .05; *t*: −0.33), and Mg (*P* > .05; *t*: 0.61) did not differ significantly between inner and outer wood (see Table 2).

**Table 2.** Summary characteristics of wood traits, of inner and outer wood, measured on 22 tree species from a lowland tropical forest in French Guiana.

Trait	Abbrev.	Unit	Inner wood				Outer wood			
			Mean	SD	Range	*n*-fold variation	Mean	SD	Range	*n*-fold variation
WSG	WSG	Unitless	0.60	0.21	0.21–0.99	4.58	0.65	0.178	0.38–0.98	2.55
Wood phosphorus concentration	Wood P	μg g^−1^	54.1	31.7	8.90–135.7	15.2	70.67	31.5	29.0–135.7	4.67
Wood calcium concentration	Wood Ca	μg g^−1^	2166.7	1719.1	281.5–7239.6	25.7	2227.8	2016.0	281.5–7239.6	25.7
Wood potassium concentration	Wood K	μg g^−1^	232.8	281.1	25.3–1379.8	54.3	172.7	154.4	19.4–643.5	33.1
Wood magnesium concentration	Wood Mg	μg g^−1^	400.8	317.0	59.9–1269.7	21.1	374.3	209.1	63.2–1269.7	20.0
Axial parenchyma fraction	*F*_AP_	Unitless	14.08	8.87	0.46–32.4	70.0	14.7	7.95	1.34–31.4	23.4
Radial parenchyma fraction	*F*_RP_	Unitless	13.7	6.46	2–32.9	16.4	15.0	7.44	2–32.9	16.41
Fibre fraction	*F*_F_	Unitless	65.15	11.60	47.47–84.36	1.77	60.75	9.37	41.28–74.81	1.81

Abbreviation (Abbrev.), mean, standard deviation (SD), range, and *n*-fold variation are shown.

### Relationships of wood nutrients with WSG, fibre fractions, and parenchyma fractions

**Figure 2.**
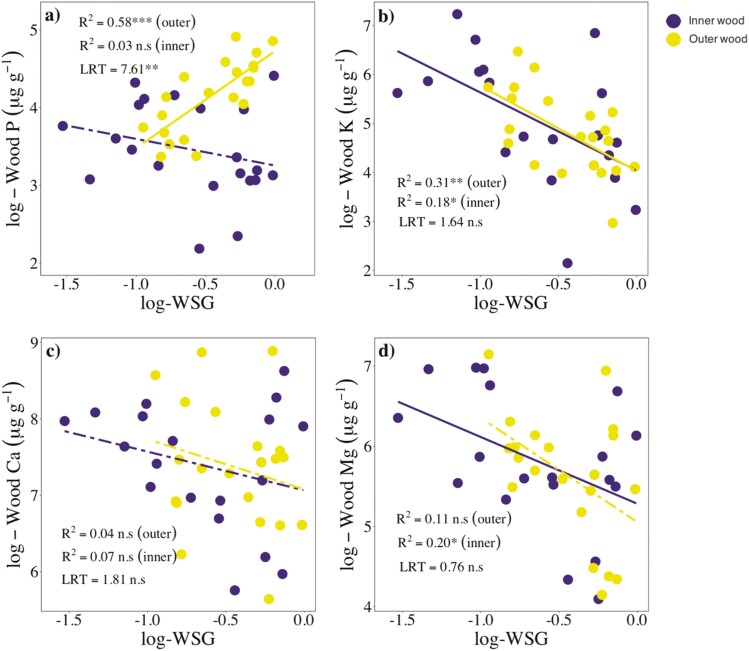
SMA regressions between WSG and wood nutrients concentrations in inner and outer wood for 22 species from a lowland forest in eastern Amazonia. Solid and dashed lines represent significant (*P* < .05) and non-significant relationships, respectively. Coefficients of determination (*R*^2^) and significance levels (^ns^*P* > .05; **P < .01; ***P ≤ .001) of the corresponding major axis regressions are shown. The differences between slopes of inner and outer wood were examined using LRT. Significant differences (*P* ≤ .05) indicate that the slopes differed.

Wood P and WSG scaled positively in outer wood (*R*^2^ = 0.58, *P* < .001), but were unrelated in inner wood (Fig. 2a). The slopes of this relationship in inner and outer wood differed significantly (LRT: 7.61; *P* ≤ .05). Wood K was negatively related to WSG in both inner (*R*^2^ = 0.18, *P* < .05) and outer wood (*R*^2^ = 0.31, *P* < .01; Fig 2b). Wood Mg decreased significantly with WSG in inner wood (*R*^2^ = 0.20, *P* < .05; Fig. 2d), and was unrelated to WSG in outer wood (Fig. 2d). In contrast, Ca concentrations were unrelated to WSG in both inner and outer wood (Fig. 2c). The slopes of the relationships of WSG with K, Ca, and Mg did not differ significantly between inner and outer wood (Fig. 2b–d).

**Figure 3.**
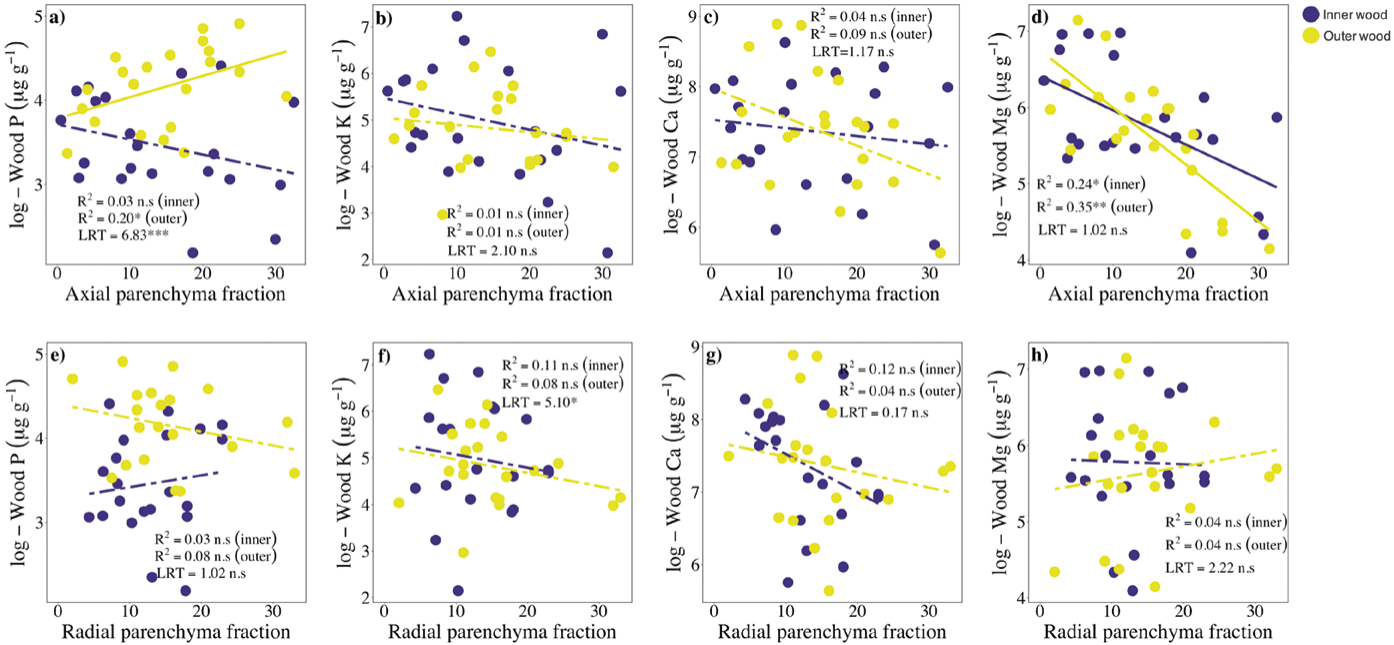
SMA regressions between wood nutrient concentrations and xylem parenchyma fractions, in inner and outer wood, across tree species (*n* = 21) from a lowland forest in eastern Amazonia. Solid and dashed lines represent significant (*P* < 0.05) and non-significant relationships, respectively. Coefficients of determination (*R*^2^) and significance levels (^ns^*P* > .05; *P < .05; **P < .01) of the corresponding major axis regressions are shown. The differences between slopes of inner and outer wood were examined using LRT. Significant differences (*P* ≤ .05) indicate that the slopes differed.

Phosphorus and axial parenchyma fractions (*F*_AP_) were positively related in outer wood (*R*^2^ = 0.21, *P* < .05; Fig. 3a), but unrelated in inner wood (Fig. 3a), and the slope of this relationship differed significantly between inner and outer wood (LRT: 6.83, *P* < .01; Fig. 3a). Potassium and Ca were unrelated to *F*_AP_ in both inner and outer wood (Fig. 2b and c); while Mg was negatively related to *F*_AP_ in inner (*R*^2^ = 0.24, *P* < .05; Fig. 2d) and outer wood (*R*^2^ = 0.34, *P* < .05; Fig. 3d), but the slope of this relationship did not differ significantly between inner and outer wood (Fig. 3d). Radial parenchyma fractions were unrelated to all wood nutrients in both inner and outer wood (Fig. 3e–h).

**Figure 4.**
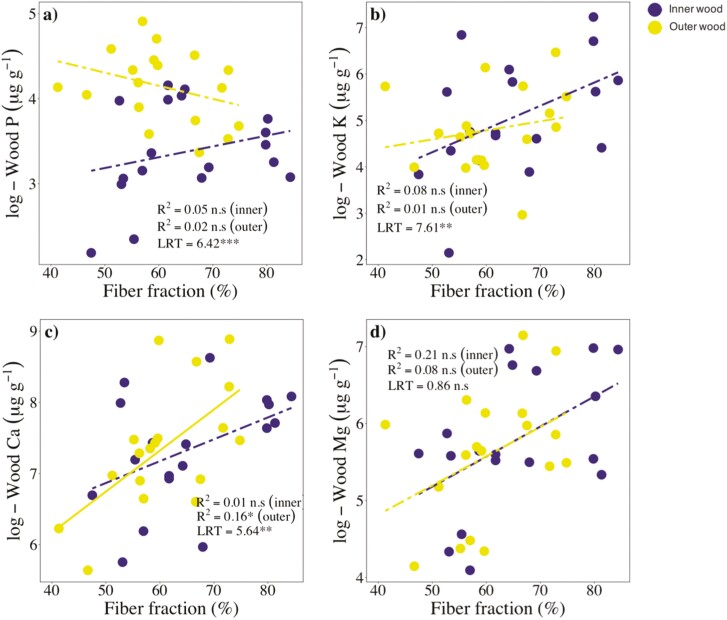
SMA regressions between wood nutrient concentrations and fibre fractions, in inner and outer wood, across tree species (*n* = 19) from a lowland forest in eastern Amazonia. Solid and dashed lines represent significant (*P* < .05) and non-significant relationships, respectively. Coefficients of determination (*R*^2^) and significance levels (n.s, P > 0.05; *, *P* ≤ 0.05) of the corresponding major axis regressions are shown. The differences between slopes of inner and outer wood were examined using LRT. Significant differences (*P* ≤ .05) indicate that the slopes differed.

Wood Ca and fibre fractions (*F*_F_) were positively related in outer wood (*R*^2^ = 0.16, *P* < .05; Fig. 4c), but unrelated in inner wood (*R*^2^ = 0.01, *P* > .05; Fig. 4c), and the slope of this relationship differed significantly between inner and outer wood (LRT: 5.64; *P* < .01; Fig. 4c). Wood P, K, and Mg were unrelated, in both inner and outer wood, to *F*_F_ (Fig. 4a, b, and d).

### Resorption of wood nutrients

**Figure 5.**
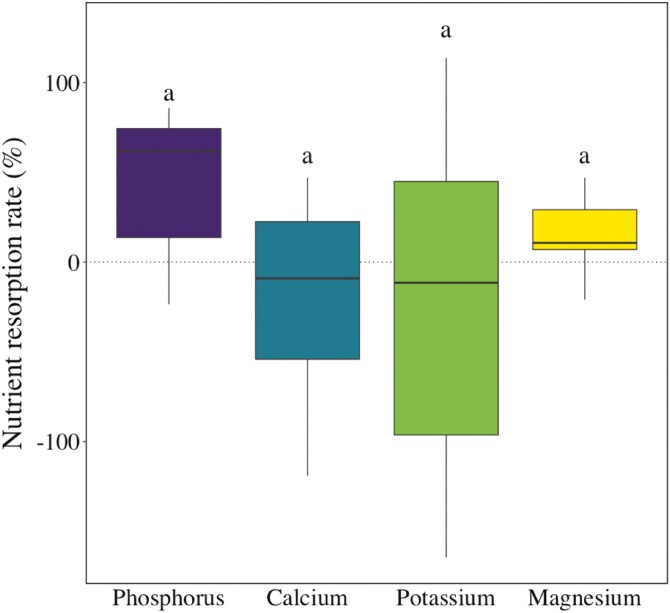
Comparison of wood nutrient resorption rates of (*n* = 14) tree species from a lowland forest in eastern Amazonia. Letters above the boxplots indicate similar (*P* > .05; Tukey’s HSD) resorption rates among nutrients.

**Figure 6.**
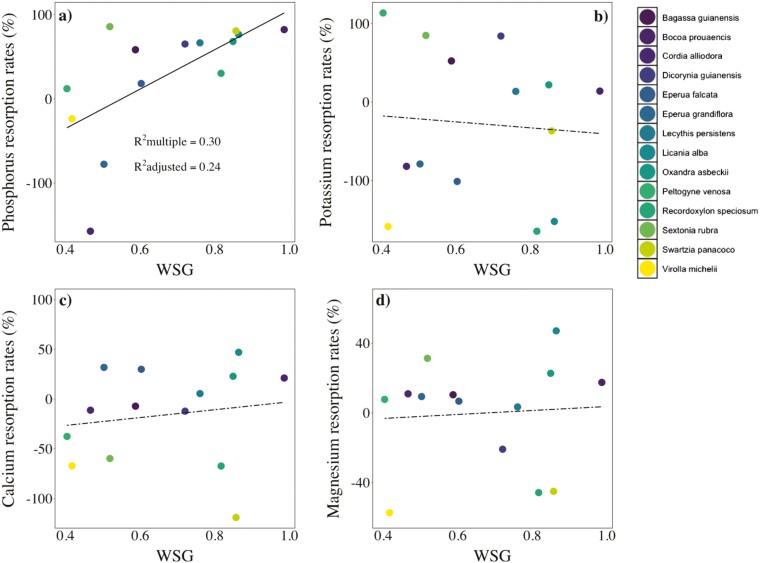
Linear models showing the relationships between nutrient resorption rates and WSG across (*n* = 14) tree species from a lowland forest in eastern Amazonia. Solid and dashed lines represent significant (*P* < 0.05) and non-significant relationships, respectively. Multiple and adjusted coefficients of determination (*R*^2^) are shown only for the significant relationship.

Resorption rates did not differ significantly among nutrients (*F* = 1.63; *P* > .05; Fig. 5), but nutrient resorption rates varied considerably among species (Fig. 6). Phosphorus resorption rates scaled significantly with WSG (*R*^2^ = 0.24; *P* < .05; Fig. 6a), while K, Ca, and Mg resorption rates were unrelated to WSG (Fig. 6b–d).

## Discussion

### Wood nutrient variation across species

Both inner and outer wood nutrient concentrations varied considerably across species. Overall, the range of wood nutrient concentrations, for inner and outer wood, we observed fell within the range of values previously reported in a meta-analysis of tropical forests (Bauters *et al.* 2021). Yet, species mean value of wood Ca tended to be higher, while values of P and K were in general lower, than those reported for other tropical forests (Bauters *et al.* 2021). Species averaged value of wood Mg concentration was lower than values for Congolese and Guianian forests, but higher than those reported for Bornean, Panamanian, and Amazonian forests (Bauters *et al.* 2021).

### Relationships of wood nutrients with WSG, fibre fractions, and parenchyma fractions

We found that wood P scaled with WSG in outer wood. This is contrary to our hypothesis and to recent studies that have reported a negative relationship between WSG and wood P (Heineman *et al.* 2016; Inagawa *et al.* 2023), and suggests that high-WSG, shade-tolerant species store higher amounts of wood P than low-WSG, fast-growing species. Wood phosphorus storage is thought to be relevant for plants growing on soils with low P availability, and particularly advantageous for shade-tolerant species, as they have longer-life cycles and would have to counteract P limitation for longer periods in comparison to most fast-growing species (Sardans and Peñuela 2014). As P plays a key role in metabolism (Jiang *et al.* 2019), and given that wood parenchyma cells are alive and metabolically active (Spicer and Holbrook 2007), a percentage of wood P would be stored and used to maintain living parenchyma cells that are involved in storage, defence, and hydraulic balance (Morris *et al.* 2016b). Moreover, some species from P-limited sites may accumulate, and not store to further use, a fraction of wood P in order to outcompete neighbouring trees (Bauters *et al.* 2022).

We also found that P was positively related to *F*_AP_ in inner wood_._ Previous studies have reported positive relationships between wood nitrogen concentrations and total (i.e. axial + radial; Merrill and Cowling 1966) or axial parenchyma fractions (Kotowska *et al.* 2020), but to our knowledge, this is the first study to report a link between *F*_AP_ and wood P concentrations. It can be difficult to separate the functions of axial and radial parenchyma cells, as they can perform simultaneously different functions and are ultimately interlinked forming a complex three-dimensional network (Morris *et al.* 2016a, b). However, some studies suggest that axial and radial parenchyma cells might be functionally different to some extent (Zheng and Martínez-Cabrera 2013; Morris *et al.* 2017). In this sense, our findings, together with those reported recently by Kotowska *et al.* (2020), suggest that axial parenchyma cells may possibly serve as a storage reservoir of mineral nutrients.

In line to our hypothesis, wood Ca was positively related to fibre fractions in outer wood. This may be explained by the fact that most Ca tends to be allocated to cell walls (Lautner *et al.* 2007), and fibres are typically the most abundant wood cell type in angiosperms, and have thicker walls than vessels or parenchyma cells (e.g. Zieminska *et al.* 2015). WSG was negatively related to wood Mg in inner wood, and to wood K in both inner and outer wood. These results differ from those of Heineman *et al.* (2016) which showed no relationships between WSG and wood K or Mg, and are in line with Lira-Martins *et al.* (2022) and Inagawa *et al.* (2023) who found a negative association between WSG and wood K. Contrary to what we expected, wood Mg was unrelated to fibre fractions, while wood K was unrelated, or negatively related, to parenchyma fractions. One possible explanation for the lack of association between wood K and parenchyma fractions would be the occurrence of living fibres, which can play an important role and storage (Carlquist 2015; Plavcová *et al.* 2016, 2023), in three of our study species, namely *S. morototoni*, *S. rubra*, and *C. obtusa*. However, we ruled out this explanation since excluding these three species from the analyses did not change the results (data not shown). Further studies using techniques such as scanning electron microscopy would be valuable to clarify how nutrients are allocated to wood cells.

A common prediction derived from the WES is that wood functional traits covary with WSG across species (Chave *et al.* 2009; Heineman *et al.* 2016). According to this expectation, we found that WSG, *F*_AP_, and wood P were positively correlated to each other in outer wood (Fig. 2; Supplementary Fig. S1 and S2). The main benefits of high WSG are suggested to be high decay resistance (i.e. Chave *et al.* 2009; Hérault *et al.* 2010) and lower stem maintenance costs (Larjavaara and Muller-Landau 2010). Moreover, high WSG is thought to increase stem hydraulic capacitance, as species with high WSG have been found to have lower sapwood water potential at turgor loss points (Santiago *et al.* 2018). Our finding that WSG, *F*_AP_, and P concentrations were positively related to each other in outer wood suggests that, at least for our study species, an additional benefit of high WSG may possibly be higher fractions of axial parenchyma cells, which may possibly favour higher storage of P. For our study species, axial parenchyma fractions are relatively low (<30%; Supplementary Fig. S1); thus, investing in large parenchyma factions may not compromise WSG. Our findings also suggest, however, that the variation of wood traits along the WES is more complex than previously assumed. Part of this complexity in wood trait covariation relates to inner and outer wood. For instance, WSG, wood P, and *F*_AP_ were related to each other in outer, but not in inner wood. This may reflect the presence of sapwood and heartwood, since a close association between traits is expected in sapwood (i.e. outer wood), which is the physiologically active part of the trunk.

### Nutrient reabsorption rates

All nutrient resorption rates we found were similar, on average, to those reported previously for temperate (Meerts 2002), as well as for Bornean (Inagawa *et al.* 2023) and Panamanian forests (Heineman *et al.* 2016) with relatively less nutrient-limited soils in comparison to Paracou. Contrary to our hypothesis that wood P or K resorption rates would be higher than those of Ca and Mg, we found similar resorption rates among nutrients when all species were pooled. This, together with the fact that there was a large interspecific variation in nutrient resorption rates, suggests that, at our study site, wood P, K, Ca, and Mg resorption rates would be mainly species-specific, and possibly influenced by species growing conditions. While we did not find significant differences in resorption rates among nutrients when species were pooled, our data suggest that P resorption at the species level tended to be more common (i.e. more positive values in Fig. 4), or pronounced (i.e. higher positive values in Fig. 4), than that of other nutrients. A more frequent or pronounced resorption of wood P might suggest a possible adaptation to soil P limitation at our study site, which may allow some species to reduce metabolic costs associated to P acquisition and be less dependent on external P supply (e.g. Urbina *et al.* 2021). The higher, or more frequent, wood P resorption may be related to functional differences among nutrients. For example, while most P is present in plants in inorganic forms and acts as a structural component of DNA and RNA, or as a metabolic energy unit in ATP (Jiang *et al.* 2019), Mg and Ca are typically found in cell walls (Meerts 2002) and thus are harder to remobilize. We also found that some species had negative nutrient resorption rates (i.e. higher nutrient concentrations in heartwood than in sapwood). This suggests that some of our study species do not need to resorb certain nutrients from woody tissues, or that a fraction of some nutrients can be deposited in heartwood and cannot be remobilized.

As we expected, phosphorus resorption rates were higher in high-WSG, shade-tolerant species. Nutrient resorption from senescing tissues is considered a key mechanism to increase their use efficiency in plant communities with poor nutrient soils (Hill 1980; Urbina *et al.* 2021), and it may be particularly important for long-lived, shade-tolerant species, since they would have to counteract soil nutrient deficiencies for a longer time in comparison to short-lived, fast-growing species (Sardans and Peñuelas 2014). Phosphorus resorption is of particular interest given that P limitation is expected to become increasingly important as a consequence of raising atmospheric CO_2_ and nitrogen deposition (Jiang *et al.* 2019). Furthermore, P resorption might have important implications for community dynamics in forests growing on P-limited soils. For example, in Paracou, soil P content is a weak predictor of biomass and demographic rates (Grau *et al.* 2016), suggesting that nutrient-cycling mechanisms, such as P resorption from woody tissues, might possibly drive forest structure and dynamics (Grau *et al.* 2016). Furthermore, in P-limited tropical forests, low soil P availability may act as a strong selective pressure favouring the dominance of species efficient at storing or resorbing P (Lovelock *et al.* 2007). As most tree species from Guianan forests are shade-tolerants (ter Steege *et al.* 2000, 2006; Molino and Sabatier 2002), we suggest that further studies should examine the possible links of wood P storage and resorption with tree species abundance in these forests.

## Conclusions

This study illustrates that wood P, K, Ca, and Mg concentrations can have different patterns of associations with WSG and wood anatomical traits, and that some of these associations change between inner and outer wood. It also suggests that high-WSG, shade-tolerant species have higher wood P concentrations in outer wood, and are more efficient at resorbing wood P than low-WSG, fast-growing species. Although these findings represent valuable insights into how wood nutrients are related to other wood traits, and how nutrient resorption rates are linked to species ecological strategies, they should be viewed with caution as they are based on a relatively small number of tree species from one study site. We suggest that future studies should examine whether the results reported here hold true for larger sets of species and other forest types.

## Supplementary Material

plaf001_suppl_Supplementary_Figures_S1-S2

## Data Availability

The data used in this manuscript are openly available in the following link: https://doi.org/10.6084/m9.figshare.20135036.v1
