# Supplementary material for: Tropical tree species with high wood specific gravity have higher concentrations of wood phosphorus and are more efficient at resorbing it
Source: AoB Plants. 2025 Jan 3;17(1):plaf001. doi: 10.1093/aobpla/plaf001 (PMC11752641; doi:10.1093/aobpla/plaf001)
Supplement: plaf001_suppl_Supplementary_Figures_S1-S2 [file plaf001_suppl_supplementary_figures_s1-s2.pdf]

Supporting information

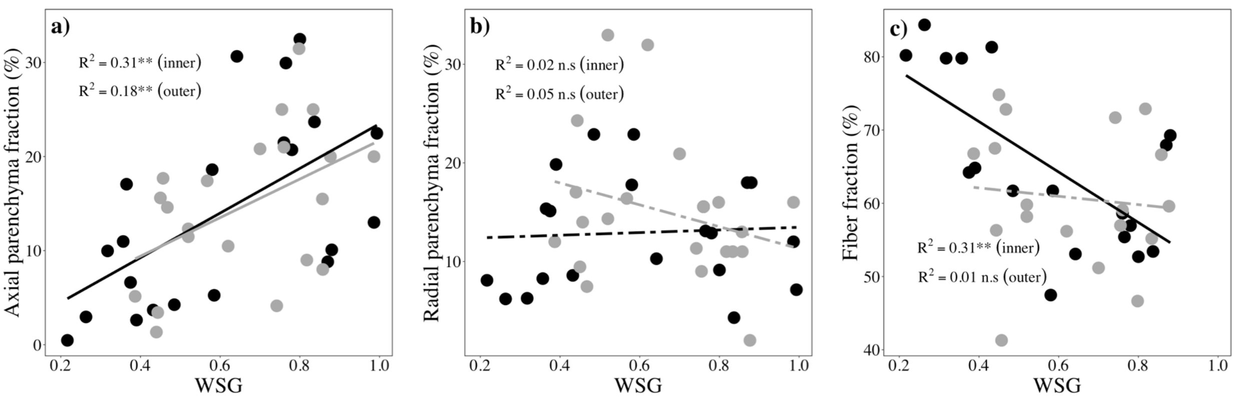

Figure S1. Linear models showing the relationships of WSG with axial parenchyma fraction (a), radial parenchyma fraction (b) and fiber fraction (c), in inner (black circles) and outer wood (gray circles), across (n=21) tree species from a lowland forest in eastern Amazonia. Solid and dashed lines represent significant (P<0.05) and non-significant relationships, respectively. Coefficients of determination (R^2) and significance levels (n.s, P=>0.05; **,P=<0.01) of the corresponding linear models are shown.

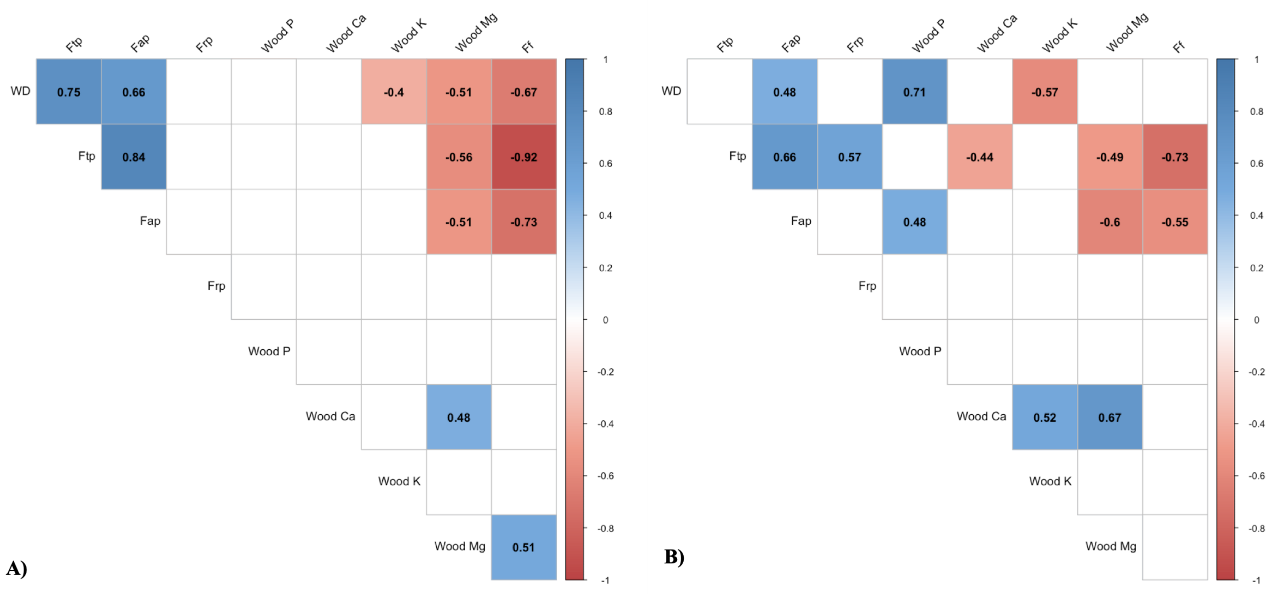

Fig. S2. Correlation matrices, using Pearson correlation coefficients, between wood traits measured on inner (A) and outer wood (B). Significant correlations (P<0.05) are colored, where blue shades represent positive correlations and red shadows negative ones. See Table 2 for trait abbreviations.
